# Gender Difference in the Relationship of Physical Activity and Subjective Happiness Among Chinese University Students

**DOI:** 10.3389/fpsyg.2021.800515

**Published:** 2021-12-07

**Authors:** Wenning Jiang, Jin Luo, Hannan Guan

**Affiliations:** ^1^Antai College of Economics and Management, Shanghai Jiao Tong University, Shanghai, China; ^2^School of International and Public Affairs, Shanghai Jiao Tong University, Shanghai, China; ^3^Institute of Healthy Yangtze River Delta, Shanghai Jiao Tong University, Shanghai, China; ^4^School of Education, Shanghai Jiao Tong University, Shanghai, China; ^5^Department of Education Information Technology, East China Normal University, Shanghai, China

**Keywords:** physical activity, happiness, gender difference, China, students

## Abstract

**Background:** Gender makes a difference in health and physical activity (PA). This research aimed to identify the gender difference in the relationship of PA and subjective happiness among Chinese university students.

**Methods:** A cross-sectional survey was conducted in Shanghai Jiao Tong University from July 7 to 17 in 2021, using an anonymous online self-report questionnaire. The questionnaire included the Chinese version of the International Physical Activity Questionnaire-Short Form (IPAQ-SF), the Subjective Happiness Scale (SHS), the Depression Anxiety Stress Scale-21(DASS-21). The demographic and health-related factors were also reported. Linear regression was carried out to identify the relationship of PA and subjective happiness.

**Findings:** In total, 1,512 students (1,108 males and 404 females) from three comprehensive schools completed the survey. The SHS score was 17.20 ± 3.44, and it was 17.00 ± 3.23 in males, and 17.75 ± 3.93 in females, respectively (*p* < 0.001). There was no gender difference in vigorous, moderate PA, or walk min/week, while female students had longer sedentary behavior hours/day than males. Male students scored higher in each subscale of DASS-21. After controlling for confounders, vigorous PA min/week (β = 0.002, *p* = 0.002) and sedentary behavior hours/day (β = 0.168, *p* = 0.005) were significantly positively associated with happiness in male students. In contrast, walk min/week (2= 0.002, *p* = 0.005) was significantly positively associated with happiness in female students.

**Interpretation:** This research demonstrated a significant gender difference in the association between PA and happiness. Policymakers and college management should pay more attention to PA programs to promote students’ happiness and mental health status.

## Introduction

Physical activity (PA) is generally defined as body movement produced by muscles resulting in energy expenditure. PA includes occupational, sports, conditioning, household, or other activities (Rhodes and Sui, 2021). The benefits of PA on mental health have been broadly explored (Zhang and Chen, 2019; Ai et al., 2021; Guan et al., 2021; Kemel et al., 2021; Zemlin et al., 2021). However, many studies focused on the effects of PA on negative emotions, for example, depression and anxiety (Ströhle, 2009) or mental disorders (Rosenbaum et al., 2014). In comparison, the relationship between PA and positive emotion has been explored insufficiently.

Happiness is a positive component of mental well-being, generally defined as subjective enjoyment and contentment (Helliwell and Aknin, 2018). Some people rank happiness as one of the most important goals of their lives (Diener and Seligman, 2004). Many studies demonstrated the health benefits of happiness. For example, happiness is associated with lower disease and mortality risk (Trudel-Fitzgerald et al., 2019; Jenkins et al., 2021).

Among the associated factors of happiness, it is believed that PA is a significant one. For example, Lathia et al. (2017) revealed that individuals with more PA are happier among 10,889 freely available app users. Another study in 15 European countries showed that walking and vigorous PA were positively associated with happiness, while moderate PA was not associated with happiness (Richards et al., 2015). Some studies indicated that the effects of PA on happiness would be a favorable research area (Zhang and Chen, 2019; van Woudenberg et al., 2020). Meanwhile, a few studies have showed that female university students are more inactive than male students, by showing longer sedentary time or lower levels of PA (Han et al., 2017; Castro et al., 2018; Zhou et al., 2021).

As gender makes a difference in PA and happiness (Drehmer, 2018; Molsted et al., 2021) and health (Stenberg et al., 2021), we hypothesize that gender difference may exist in the correlation between PA and happiness. According to our best knowledge, rare studies focused on the gender difference in the association of PA and happiness. Therefore, this study aimed to explore the association of happiness with gender and PA in university students.

## Materials and Methods

### Study Design and Participants

A cross-sectional survey was conducted in Shanghai Jiao Tong University from July 7 to 17 in 2021. Three comprehensive schools were chosen as convenience samples, and all undergraduates in these schools were invited to complete an anonymous online self-administered questionnaire. The QR code and weblink of the survey were posted online via WeChat. Generally, it took the participants 5–8 min to complete the questionnaire.

The Ethics Committee in Shanghai Jiao Tong University approved the research protocol (approval number: H2021158I). Each participant obtained the consent form before the response to the questionnaire.

### Measures

#### Socio-Demographic Characteristics

This part involved essential socio-demographic characteristics of university students, which were selected based on previous studies (Ráthonyi et al., 2021; Yang et al., 2021; Zhou et al., 2021), including age, gender, grade, place of hometown, ethnic groups, specialty, monthly allowances, marital status.

#### Physical Activity

PA was assessed by the Chinese version of the International Physical Activity Questionnaire-Short Form (IPAQ-SF), which has adequate validity and reliability (Mengyu, Fan et al., 2014; Meh et al., 2021). The participants reported days and times of PA during the last 7 days. PA was divided into three intensities: vigorous, moderate, and walking. Participants also reported sedentary behavior hours of each day. The Chronbach’s α of IPAQ-SF was 0.73 in this study.

#### Subjective Happiness

We measured subjective happiness through the Chinese version of the Subjective Happiness Scale (SHS), which contains four items about subjective happiness (Lyubomirsky and Lepper, 1999). Previous studies demonstrated the Chinese version of SHS had substantial reliability and validity for subjective happiness in general Chinese population (Nan et al., 2014; Guo et al., 2020). Students were asked to respond to a Likert scale ranging from 1 (strongly disagree) to 7 (strongly agree). The total score of subjective happiness was computed by summing all four items, while the second and the fourth items were reverse-coded. The Chronbach’s α of SHS in our samples was 0.79.

#### Other Related Factors

Meanwhile, we used a Chinese version of the Depression Anxiety Stress Scale-21 (DASS-21) to assess depression, anxiety, and stress level, according to the same cut-off values reported previously (Jiang et al., 2020). The Chronbach’s α of DASS-21 was 0.96. Alcohol use and cigarette and frequency of insomnia were also reported.

### Data Analysis

We used one-sample K-S test to examine the normality of obtained data. Descriptive analyses about the sample’s socio-demographic, PA and sedentary behavior times, happiness level, depression, anxiety, stress status, and other related factors were conducted. Demographic, PA, happiness, and related variables of male and female students were compared using *t-*test or Mann-Whitney *U*-test for continuous variables and the chi-squared test for categorical variables. We used backward stepwise multiple regression analysis using subjective happiness level as the dependent variable to assess the effects of variables including PA, sedentary behavior, demographic, and other related factors in male and female students.

All statistical analyses were performed through the STATA software version 16.0 (Stata Corporation, College Station, TX, United States), with the significance level at the *p*-value of 0.05 (two-tailed).

## Results

### Sample Characteristics

In total, 4,561 students were invited to participate, and 1,534 students responded (response rate = 33.63%). Finally, 1,512 students completed the questionnaire without logical errors and were included in the statistical analysis. Their mean age was 21.20 ± 4.25 years, and 73.28% of them were male. The demographic characteristics and related data of male and female students are shown in Table 1. Compared with female students, male students had higher depression, anxiety, stress level and were more likely to use cigarettes/alcohol.

**TABLE 1:** Characteristics of participants by gender, *n* (%).

Characteristic		Total (1512)	Male (1108)	Female (404)	*p*
Grade					**0.027**
	Freshman	406 (26.85)	311 (76.60)	95 (23.40)	
	Sophomore	408 (26.98)	307 (75.25)	101 (24.75)	
	Junior	463 (30.62)	316 (68.25)	147 (31.75)	
	Senior	235 (15.54)	174 (74.04)	61 (25.96)	
Place of hometown					0.254
	Urban	993 (65.67)	737 (74.22)	256 (25.78)	
	Rural	519 (34.33)	371 (71.48)	148 (28.52)	
Ethnicity					0.060
	Han Chinese	1,388 (91.80)	1,026 (73.92)	362 (26.08)	
	Minority nationality	124 (8.20)	82 (66.13)	42 (33.87)	
Monthly allowances (RMB)					0.145
	<1,000	188 (12.43)	141 (75.00)	47 (25.00)	
	1,000–1,499	511 (33.80)	379 (74.17)	132 (25.83)	
	1,500–1,999	395 (26.12)	294 (74.43)	101 (25.57)	
	2,000–2,499	231 (15.28)	172 (74.46)	59 (25.54)	
	2,500–2,999	66 (4.37)	40 (60.61)	26 (39.39)	
	≥3,000	121 (8.00)	82 (67.77)	39 (32.23)	
Relationship status					0.387
	Not dating nor married	870 (57.54)	627 (56.59)	243 (60.15)	
	Dating but unmarried	537 (35.52)	398 (35.92)	139 (34.41)	
	Married	64 (4.23)	52 (4.69)	12 (2.97)	
	Others	41 (2.71)	31 (2.80)	10 (2.48)	
Insomnia					0.983
	No	630 (41.67)	465 (73.81)	165 (26.19)	
	Seldom (≤3 times/month)	451 (29.83)	327 (72.51)	124 (27.49)	
	Sometimes (1–2 times/week)	280 (18.52)	205 (73.21)	75 (26.79)	
	Often (3–5 times/week)	113 (7.47)	84 (74.34)	29 (25.66)	
	Daily	38 (2.51)	27 (71.05)	11 (28.95)	
Cigarette use					**<0.001**
	No	1187 (78.51)	822 (69.25)	365 (30.75)	
	Ex-smoker	190 (12.57)	162 (85.26)	28 (14.74)	
	Current smoker	135 (8.93)	124 (91.85)	11 (8.15)	
Alcohol use					**<0.001**
	Never	874 (57.80)	575 (65.79)	299 (34.21)	
	Sometimes (1–4 times/month)	559 (36.97)	470 (84.08)	89 (15.92)	
	Often (>4 times/month)	79 (5.22)	63 (79.75)	16 (20.25)	

		**Mean ± SD**	**Mean ± SD**	**Mean ± SD**	***p***

Age (years)		21.20 ± 4.25	21.27 ± 4.27	21.00 ± 4.19	0.273
DASS-Depression		27.03 ± 9.82	27.51 ± 9.82	25.73 ± 9.71	**<0.001**
DASS-Anxiety		26.21 ± 9.72	26.73 ± 9.69	24.80 ± 9.67	**<0.001**
DASS-Stress		23.59 ± 8.11	23.84 ± 8.10	22.89 ± 8.10	**0.022**

*Bold value for p < 0.05.*

Male students reported having 6.21 ± 1.53 sedentary behavior hours/day, while female students had 6.44 ± 1.74 h/day, which was significantly higher. Meanwhile, female students have a higher level of happiness than males. There were no significant differences in times of PA between male and female students. Table 2 shows the detailed characteristics.

**TABLE 2:** Subjective happiness and physical activity of participants by gender.

	Total (mean ± SD)	Male (mean ± SD)	Female (mean ± SD)	*p*
SHS score	17.20 ± 3.44	17.00 ± 3.23	17.75 ± 3.93	**<0.001**
Sedentary behavior hours/day	6.27 ± 1.59	6.21 ± 1.53	6.44 ± 1.74	**0.006**

	**Mean (median, IQR)**	**Mean (median, IQR)**	**Mean (median, IQR)**	

Vigorous PA min/week	37.27 (0.0)	37.73 (0.0)	36.01 (0.0)	0.759^a^
Moderate PA min/week	50.32 (0.0)	47.25 (0.0)	58.74 (0.0)	0.345^a^
Walk min/week	108.92 (0.0)	106.38 (0.0)	115.92 (0.0)	0.556^a^

*Bold value for p < 0.05; IQR, inter-quartile range. ^a^Mann-Whitney test.*

### Association of Physical Activity and Subjective Happiness

We used backward stepwise multiple linear regression analysis for further testing. Three kinds of PA activity minutes per week and sedentary behavior hours per day were purposely remained in the regression analysis.

In male students, monthly allowance (β = 0.245, *p* < 0.001) and DASS-Depression score (β = −0.110, *p* < 0.001) were independently associated with the subjective happiness level. Among PA and sedentary behavior times, only vigorous PA min/week (β = 0.002, *p* = 0.002) and sedentary behavior hours/day (β = 0.168, *p* = 0.005) were significantly positively associated with subjective happiness. These factors together explained a 14.85% variance in the subjective happiness level.

In female students, only DASS-Depression score (β = −0.165, *p* < 0.001), rural hometown (β = −0.882, *p* = 0.016) and walk min/week (β = 0.002, *p* = 0.005) were independently associated with the happiness level. These factors explained a 21.37% variance in subjective happiness (Table 3).

**TABLE 3:** Association of subjective happiness and PA.

	Male				Female			
Subjective happiness	β	95% CI (Lower)	95% CI (Upper)	*p*	β	95% CI (Lower)	95% CI (Upper)	*p*
**PA**								
Vigorous PA min/week	0.002	0.001	0.004	**0.002**	0.001	−0.002	0.004	0.614
Moderate PA min/week	0.000	–0.002	0.001	0.605	0.000	−0.003	0.002	0.896
Walk min/week	0.000	0.000	0.001	0.465	0.002	0.001	0.003	**0.005**
Sedentary behavior hours/day	0.168	0.051	0.284	**0.005**	0.075	−0.124	0.273	0.459

**Other factors**								
Monthly allowances	0.245	0.113	0.378	**<0.001**				
DASS-depression	−0.110	–0.129	−0.092	**<0.001**	−0.165	−0.201	−0.130	**<0.001**
Place of hometown (Rural)					−0.882	−1.602	−0.163	**0.016**

*Bold value for p < 0.05.*

## Discussion

This research was one of the first to compare the gender differences in the relationship between PA and subjective happiness among Chinese university students. Multivariate regression analyses showed that vigorous PA min/week and sedentary behavior hours/day were significantly associated with happiness in male students. In contrast, walk min/week was associated with happiness in female students. This research demonstrated that there was a significant gender difference in the association between PA and happiness.

Gender differences significantly impact depression, anxiety, and stress levels in this study, as male students have a higher score of depression, anxiety, and stress. This is an intriguing finding against several previous studies in other countries. Ochnik et al. (2021) surveyed 2,349 students in nine countries through Generalized Anxiety Disorder (GAD-7), Perceived Stress Scale (PSS-10), to demonstrate that female was a credible predictor for GAD-7 and PHQ-8 scores. Another survey in 1,224 Brazilian university students also showed that female predictors for symptoms of depression, anxiety, and stress according to DASS-21 (Lopes and Nihei, 2021). A study in 515 Malaysian university students demonstrates that female students scored significantly higher in DASS-21 (Pang et al., 2021). This kind of difference may be due to cultural factors and role expectations. In China, males generally have more aggressive norms and expectations than females, while females are more demanding for their household management than males (Zhou, 2018; Liu et al., 2021).

This study found that vigorous physical activity was significantly associated with subjective happiness in male students, and walk was associated with females. It suggests that male students should spend more time in vigorous PA and females more walking for optimal mental health. The relationship between PA and happiness has been widely documented (Zhang and Chen, 2019; Le et al., 2021). In most studies, the intensity of the physical activities had not been distinguished in the relationship. Furthermore, another research showed that moderate-intensity physical activity was not associated with happiness (Richards et al., 2015), which is aligned with this study. Meanwhile, Fisher et al. (2019) found out that vigorous-intensity PA was positively associated with happiness among female first-year medical students but not males, which is contrary to this study. In the future, more robust research is needed to determine the relationship.

The gender differences in the relationship could be explained by biological factors such as dopamine. As the dopaminergic system plays essential roles in many brain functions, including rewards and happy mood, it is hypothesized that dopaminergic signals act to regulate PA and happiness (Marques et al., 2021). Several studies suggested gender differences in microcircuit regulatory mechanisms, which can change dopamine dynamics (Hasbi et al., 2020; Robinson and Banks, 2021; Shin et al., 2021; Zachry et al., 2021). Nonetheless, the effects of dopamine on PA and happiness were unclear. More studies are needed to clarify the mechanism of gender differences in the association of PA and happiness.

This study has several limitations. First, as a cross-sectional survey, the causal relationship between PA and happiness cannot be addressed. Second, this study did not control factors such as family income, social status, and Body Mass Index, which can affect the relationship between PA and happiness. Third, all students were recruited from Shanghai Jiao Tong University in Shanghai, China. As a result, the generalizability of the research conclusions is limited. Fourth, the recall bias and response bias cannot be ruled out in this study.

## Conclusion

The current study demonstrated that vigorous PA was significantly associated with happiness in male students, while walk was associated with happiness in female students. Policymakers and college management should pay more attention to PA programs to promote students’ happiness and mental health status.

## Data Availability Statement

The raw data supporting the conclusions of this article will be made available by the authors, without undue reservation.

## Ethics Statement

The studies involving human participants were reviewed and approved by the Ethics Committee in Shanghai Jiao Tong University. The patients/participants provided their written informed consent to participate in this study. Written informed consent was obtained from the individual(s) for the publication of any potentially identifiable images or data included in this manuscript.

## Author Contributions

JL made substantial contributions to the study design and critically revised the manuscript. All authors have read and approved the published version of the manuscript.

## Conflict of Interest

The authors declare that the research was conducted in the absence of any commercial or financial relationships that could be construed as a potential conflict of interest.

## Publisher’s Note

All claims expressed in this article are solely those of the authors and do not necessarily represent those of their affiliated organizations, or those of the publisher, the editors and the reviewers. Any product that may be evaluated in this article, or claim that may be made by its manufacturer, is not guaranteed or endorsed by the publisher.
